# Accurate low-dose exposure assessment of benzene and monoaromatic compounds by diffusive sampling: sampling and analytical method validation according to ISO 23320 for *radiello*^®^ samplers packed with activated charcoal

**DOI:** 10.3389/fpubh.2023.1271550

**Published:** 2023-11-13

**Authors:** Laura Zaratin, Caterina Boaretto, Riccardo Carnevale Schianca, George Hinkal, Elena Grignani, Danilo Cottica

**Affiliations:** ^1^Environmental Research Centre, Istituti Clinici Scientifici Maugeri SpA SB, Perarolo di Vigonza, Italy; ^2^Concawe, Brussels, Belgium

**Keywords:** benzene, inhalation exposure assessment, sampling and analytical method validation, gasoline vapors, hexane, isopropyl benzene, diffusive sampler validation ISO 23320

## Abstract

The research study aimed at providing an accurate low-dose benzene exposure assessment method, by validating diffusive monitoring techniques for benzene personal exposure measurements at workplaces where benzene concentrations are expected in the low ppb range, such as in the present-day chemical, petrochemical, foundry, and pharmaceutical industry. The project was aimed at addressing the need for a robust and fully validated method to perform personal exposure measurements considering that the occupational exposure limit value for benzene is going to be significantly lowered in the next few years. Diffusive sampling offers a reliable alternative to pumped sampling methods, intrinsic safety in potentially explosive atmospheres, lightness, and ease of use. In this study, the *radiello*® diffusive sampler, with the packed activated charcoal RAD130 adsorbing substrate [suitable for solvent desorption and analysis by high-resolution gas chromatography-flame ionization detection (HRGC-FID)], was used. The experiments have been conducted following the ISO 23320 standard in the range from 0.005 to 0.1 ppm (16 to 320 μg/m^3^), yielding a full validation of the sampling and analytical method. The sampler performances have fulfilled all requisites of the ISO 23320 standard, in particular: bias due to the selection of a non-ideal sorbent is lower than 10% (no significant back diffusion of benzene due to concentration change in the atmosphere); bias due to storage of samples for up to 2 months is lower than 10%; nominal uptake rate for benzene on RAD130 is 74.65 mL/min; and expanded uncertainty of the sampling and analytical method is 20.6%. The sampling and analytical method is therefore fit-for-purpose for the personal exposure measurements aimed at testing compliance with occupational exposure limit values for benzene. The method is also fit for short-duration exposure monitoring related to specific tasks, and other volatile organic compounds, usually found in the same workplaces, such as aliphatic and aromatic hydrocarbons and some oxygenated compounds, have also been studied. In particular, n-hexane and isopropyl benzene, whose classification is currently under revision, can be efficiently monitored by this technique.

## Introduction

Benzene has long been recognized as a hazardous chemical agent, a Category 1A carcinogen (H350) and a Category 1B mutagen (H340) in accordance with Article 2 (b) of Directive 98/24/EC (1) and Article 2 (a) and (b) of Directive 2004/37/EC (2), respectively. The 2004 directive set the 8-h time-weighted average (8 h-TWA) occupational exposure limit value (OELV) for benzene to 1 ppm (which corresponds to 3.25 mg/m^3^ at 293 K and 1,013 hPa). A “skin notation” is applied indicating that there is a substantial contribution to the total body burden possible via dermal exposure. In 2018, the European Chemicals Agency Risk Assessment Committee (ECHA RAC) adopted an opinion to set the benzene occupation exposure limit to 0.05 ppm by considering a weight of evidence-based estimated human LOAEC (lowest-observed-adverse-effect concentration) of 1 ppm for chromosomal damage in peripheral lymphocytes of workers, acknowledging an animal LOAEC of 1 ppm for chromosomal damage in peripheral lymphocytes and bone marrow, and using assessment factors (AF) following ECHA Guidance to account for uncertainties (3). Subsequently, the Working Party of Chemicals conducted an impact assessment and the Advisory Committee on Safety and Health at Work (ACSH) revised the acceptable safe levels of workplace benzene exposure (4). In 2022, Directive (UE) 2022/431 (5) specified a new limit value for occupational exposure to benzene to improve the protection of workers from the risks related to exposure to carcinogens and/or mutagens at work. The 8 h-TWA OEL was lowered from 1 ppm to 0.2 ppm (0.66 mg/m^3^) with the following transitional measures: the limit value is 1 ppm until 5 April 2024; it will decrease to 0.5 ppm (1.65 mg/m^3^) from 5 April 2024 until 5 April 2026; and it will be further decreased to 0.2 ppm thereafter. In some EU Member States, lower OEL values, additional short-term exposure limits (STEL), or biological limit values (BLV) are also applied.

Notably, in parallel to this process, the industrial world also acknowledged the necessity to independently reassess the benzene OELV. North et al. conducted an exhaustive literature review and evaluated the molecular modes of action (MoA) in benzene-driven pathologies (6). These data led to a MoA-based threshold proposal of 0.25 ppm (8 h-TWA) which the authors consider to be associated with no significant residual cancer risk and also avoiding other adverse effects (7).

Regardless, benzene is an essential chemical intermediate in many industrial syntheses including the manufacture of plastics, dyes, detergents, pharmaceuticals, and pesticides. Benzene, as a monoconstituent (CAS No 71–43-2), has over 100 active registrations as of 2022 under EU-REACH. Benzene is also a constituent/impurity in many substances. Presently, there are over 100 registered substances that have a benzene content in a range of 0.1–1.0% w/w, and approximately 100 substances that have a benzene content of higher than 1.0% w/w; mainly, these substances are gasoline, naphtha, distillates from coal tar, or other types of hydrocarbon mixtures.

Workers are primarily subject to benzene exposure at the workplace in the petroleum, metal foundry, chemical, and pharmaceutical industries. Between 1950 and 1960, occupational exposure to benzene was high with estimated typical benzene concentrations between 10 and 100 ppm (32–320 mg/m^3^) or even higher than 100 ppm (320 mg/m^3^), as reported in the first epidemiological study conducted in the United States by NIOSH (the Ohio Pliofilm rubber workers cohort) (8). More recent reports and literature reviews show that benzene concentration at the workplace is constantly decreasing. Recent publications confirm that occupational exposures to benzene in the EU are usually below 1 ppm (3.25 mg/m^3^) (9, 10). Studies performed in the oil refining industry in Italy and Sweden (11, 12) showed that workplace exposure is lower than 0.2 mg/m^3^ (0.06 ppm) for refinery employees during normal plant operation. Another Swedish study (13) delved deeper into specific worker scenarios and demonstrated that personal exposure to benzene in the oil refinery sector ranged from 0.15 to 1.2 mg/m^3^ (0.05 ÷ 0.4 ppm) during plant maintenance operations.

Many other studies could be cited that lead to the conclusion that personal exposure to benzene by inhalation at the workplace is generally decreasing, mainly due to lower benzene content in the process stream, use of best available technologies, and changes in industrial practice aimed at limiting exposure, for example, the introduction of mandatory vapor recovery devices and procedures in bulk gasoline distribution operations.

While industrial practice has reduced benzene exposure, the decreased OEL implies personal monitoring in a lower exposure concentration range to assure a safe working environment. Moreover, there is a need for high-quality data to perform reliable studies on benzene low-dose exposure and its possible health effects to support any dose-related considerations. In particular, the shape of low-concentration dose–response functions and their implications for human health risk assessment should be investigated in order to investigate the hypothesis of non-linear dose–response relationships at low exposure concentrations. In fact, whereas previous studies were based on extrapolated data (14), the aim of this study was to develop a validated standard method that allows monitoring of benzene covering the range from 0.0003 to at least 0.1 ppm (i.e., from 0.001 to at least 0.3 mg/m^3^) over an 8-h exposure duration, tested according to the proper and more recent European standards and to generate high-quality data in a low-dose exposure range. The study has been developed by the Environmental Research Center of Istituti Clinici Scientifici Maugeri SpA SB and was aimed at producing a fully validated sampling and analytical method fit for obtaining reliable personal exposure data for the oil and gas, foundry, chemical, and pharmaceutical industry employees.

Diffusive sampling has been used as it can offer a dependable alternative to pumped sampling methods, with greater reliability and feasibility. Diffusive samplers, moreover, offer intrinsic safety in potentially explosive atmospheres, lightness, and ease of use for workers and industrial hygiene operators. Furthermore, they are suitable for measurement times longer than 8 h (up to 12-h shift times) because they do not need any power source to be operated. Benzene air concentration is currently measured in diverse environmental settings by diffusive techniques, for example, by the radial symmetry diffusive sampler *radiello*®, developed and produced by the Environmental Research Center of Istituti Clinici Scientifici Maugeri, Padova, Italy. In particular, two alternative configurations of the sampler can be used for workplace monitoring of benzene (along with a number of other gasoline constituents, such as aliphatic, cycloaliphatic, and aromatic hydrocarbons, and some oxygenated compounds):

(a) RAD130, packed with activated charcoal, suitable for solvent desorption with carbon disulfide and analysis by high-resolution gas chromatography coupled with flame ionization detection or mass spectrometric detection;
(b) RAD145, packed with graphitised charcoal, suitable for thermal desorption by a two-stage thermal desorption apparatus and analysis by high-resolution gas chromatography coupled with flame ionization detection or mass spectrometric detection.

The proposed radial symmetry diffusive sampling devices have been already used for environmental, indoor, and workplace monitoring, showing high sensitivity in comparison with axial diffusive samplers, which are usually characterized by lower uptake rate values (15–19). The current applicable international standard for the validation of a diffusive sampling method is EN ISO 23320:2022 (20). The activated charcoal sampler RAD130 had been validated for workplace monitoring before the EN ISO 23320:2022 standard was issued, but validation was performed in a concentration range higher than the presently addressed one. RAD145, on the other hand, had been validated in the proper measurement range for indoor air quality measurements upon exposure time durations of 4.5 to 7 days, but not as extensively as needed for complying with the EN ISO 23320:2022 standard when exposure times of 8 h are concerned. The two sampling and analysis methods were originally developed in compliance with Health and Safety Executive (HSE-UK) Methods for the Determination of Hazardous Substances MDHS88 (21) and MDHS80 (22), respectively.

The full validation of the RAD130 sampler, for benzene monitoring covering the range from 0.0003 to at least 0.1 ppm^1^ (0.001 to at least 0.3 mg/m^3^) over an 8-h exposure, is described here. The proposed method is tested to demonstrate whether it is fit for the generation of high-quality data concerning personal monitoring in a low-dose occupational exposure range in the chemical, petrochemical, foundry, and pharmaceutical industries, whereas previous studies were based on extrapolated data. The relevant novelty consists in the method being fit for the purpose of benzene exposure measurements in workplaces where its concentration is expected to be in the low ppb range. The method relies upon radial geometry diffusive samplers, which ensure adequate sensitivity, simplicity of use for industrial hygiene and HSE operators, and intrinsic safety in potentially explosive atmospheres. The samplers are very lightweight and negligibly encumbering operators even when busy with demanding tasks in the production plants. Additionally, uncertainty measurement calculations and results, and a summary of experimental results concerning other gasoline constituents, such as n-hexane, toluene, ethylbenzene, xylene isomers, and isopropyl benzene, are provided. Validation results have to be compared with the performance criteria established by the UNI EN 482:2021 Standard (23), in order to verify that the proposed sampling and analytical method is fit for obtaining personal exposure measurements to be compared with OELVs. In order to achieve monitoring methods reliable for low-dose exposure studies, the entire experimental procedure has been developed by assuming a reference value (RV) for benzene of 0.05 ppm (0.16 mg/m^3^ or 160 μg/m^3^), coinciding with the concentration value proposed by RAC in 2018. The employed analytical method (solvent desorption and high-resolution gas chromatography with flame ionization detection [HRGC-FID] for RAD130) has been accredited within our facility (Environmental Research Center of Istituti Clinici Scientifici Maugeri IRCCS Spa SB) according to ISO IEC 17025 Standard (24).

## Materials and equipment

The *radiello*® diffusive sampler employed in all experimental tests described in the present study is commercially available from Istituti Clinici Scientifici Maugeri SpA SB and is composed of:

- A stainless-steel net cylinder packed with 35–50 mesh activated charcoal (the adsorbing substrate), stored (before and after actual exposure) in a glass tube capped by a polyethylene stopper (RAD130)
- A cylindrical diffusive membrane made of microporous polyethylene (RAD120)
- A supporting plate made of polycarbonate (RAD121)
- A vertical adapter used for personal exposure in order to make the sampler less encumbering for the worker (RAD122).

Exposure is performed by inserting the charcoal adsorbing substrate, contained in the stainless-steel cylinder, into the diffusive membrane, and screwing it on the vertical adapter, already positioned onto the supporting plate. Exposure is completed when the diffusive membrane is unscrewed from the supporting plate and the adsorbing substrate is placed in the glass tube, capped with the PE stopper, and identified by the provided barcode label.

**Figure 1:**
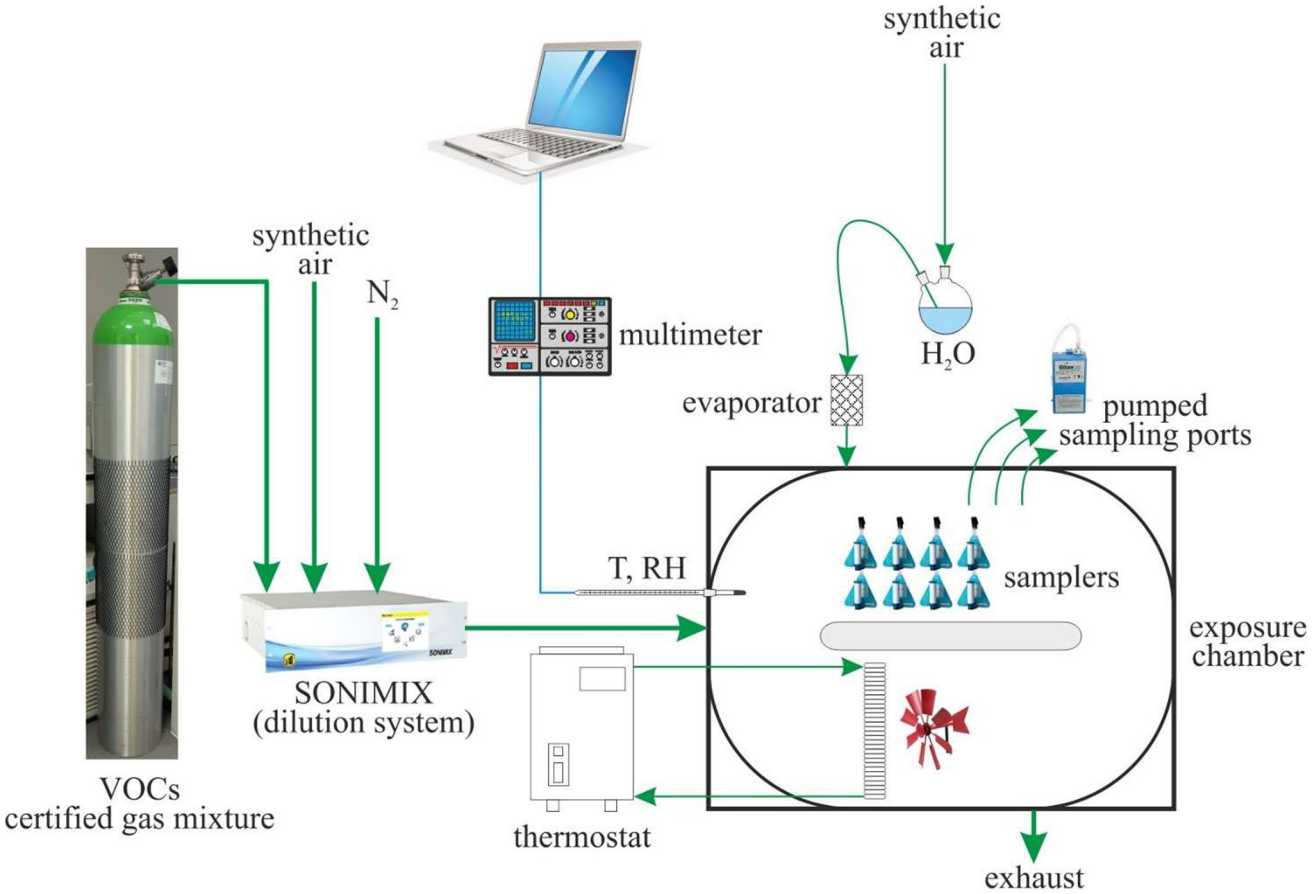
Experimental exposure chamber set-up.

Experimental tests are performed in an exposure chamber that allows the simultaneous exposure of up to 24 diffusive samplers at a time. The chamber, shown in Figure 1, is an air-tight container inside which the airflow follows a ring-shaped path, equipped with suitable devices for control of the air’s concentration level, temperature, relative humidity, and velocity. Air velocity is controlled by two fans installed inside the chamber that allow air velocities ranging from 0.5 m·s^−1^ to 2 m·s^−1^. The inner walls of the chamber are lined with polytetrafluoroethylene, and the upper lid is made of glass. The gas mixture continuously delivered to the exposure chamber is produced by dilution of a certified gas mixture (purchased by Nippon Gasses Belgium NV) containing benzene and 24 other volatile organic compounds (VOCs) at the concentrations shown in Table 1. The samplers, therefore, are simultaneously exposed to benzene and to other VOCs usually encountered at the oil and gas workplace. The mixture is diluted by a two-stage dilution system composed of critical orifices and mass flow controllers (customized design and production by LNI Swissgas, Versoix, Switzerland), which is fit for dynamic generation of gas mixtures of different concentration levels from a single cylinder, providing dilution ratios of 1:100 to 1:10,000. The dilution system is calibrated by the manufacturer, which is an ISO 17025-accredited laboratory for the measurement of gaseous flow rates. Dilution gasses are research-grade nitrogen and synthetic air purchased from SOL SpA, Italy.

**Table 1:** Composition of the certified gaseous mixture.

Component	CAS number	Concentration (ppm)
benzene	71–43-2	5.92
toluene	108–88-3	4.96
ethylbenzene	100–41-4	4.96
m-xylene	108–38-3	4.99
p-xylene	106–42-3	4.97
o-xylene	95–47-6	4.96
isopropyl benzene	98–82-8	0.99
1,3,5-trimethybenzene	108–67-8	1.01
1,2,4-trimethybenzene	95–63-6	1.01
1,2,3-trimethybenzene	526–73-8	1.00
2-methylpentane	107–83-5	5.07
3-methylpentane	96–14-0	4.93
n-hexane	110–54-3	5.02
n-heptane	142–82-5	4.99
n-octane	111–65-9	4.99
n-nonane	111–84-2	5.08
n-decane	124–18-5	4.97
n-undecane	1,120-21-4	1.01
n-dodecane	112–40-3	1.00
cyclopentane	287–92-3	4.93
methylcyclopentane	96–37-7	5.05
cyclohexane	110–82-7	5.07
methylcyclohexane	108–87-2	5.08
tert-butyl methyl ether	1,634-04-4	4.99
tert-butyl ethyl ether	637–92-3	5.00

Water vapor is directly introduced in the chamber, following a separate line with respect to the VOC-gas mixture. Liquid water, contained in a pressurized reservoir, is pushed through an evaporator and then into the chamber. The relative humidity in the chamber can be regulated from 5 to 95%. It is continuously monitored by a capacitive probe Rotronic, Hygroclip SC04 (combined humidity and temperature sensors).

Temperature is controlled by two finned heat exchangers. The temperature of the liquid flowing through the heat exchangers is regulated by a Heto HMT 200 thermostat with a CBN 8–30 recirculating bath that allows generating air temperature in the chamber from −5°C to 40°C. A Pt100 probe, Hygroclip SC04, continuously monitors temperature. Humidity and temperature probes are calibrated against a Delta Ohm HD21AB17 instrument (for relative humidity: capacitive probe, 0.1% resolution, ±2% accuracy; for temperature: NTC thermocouple, resolution 0.1°C, accuracy ±0.2°C). Humidity and temperature probes are connected to a Keithley multimeter, and data are recorded continuously (one reading every 10 s) and then processed in order to obtain average temperature and relative humidity over each exposure duration.

## Methods

Analysis of RAD130 is performed by solvent desorption, and the provided glass tube is used for extracting the sample. This is accomplished by adding to the sample 2.0 mL of carbon disulfide (low benzene content, Honeywell cat. 342,270) directly into the tube, followed by 100 μL of internal standard solution (2-fluorotoluene, purity ≥99.9% Aldrich cat. F15323, diluted to 0.1 mg/mL with carbon disulfide). The analysis is accomplished by HRGC with split mode injection and FID, with an HP-PONA column (Agilent J&W 19091S-001) or equivalent, 50 m long, with 200 μm internal diameter and 0.5 μm film thickness. Calibration standards are prepared by weighing and diluting known amounts of high-purity benzene (≥ 99.9%, Sigma-Aldrich cat. 12,540) with carbon disulfide in the concentration range from 0.05 to 25 μg/mL (the calibration curve is prepared also for other VOCs, in the range from 0.16 to 80 μg/mL). Analytical recovery tests have been performed at three loading levels for benzene and all the other VOCs; a summary of the results is displayed in Table 2. Analytical recovery is well above the minimum value of 75% for all components at all levels, with a coefficient of variation lower than 10% (as required in paragraph 6.3.2.1 of ISO 23320 standard). Anyway, the calibration procedure is always performed, in our laboratory, by phase-equilibrium method: 2.0 mL of each calibration solution is added to a blank RAD130, followed by 100 μL of internal standard solution, and then, the same procedure is applied as to the actual samples. Therefore, analytical recovery values are already taken into account.

**Table 2:** Analytical recovery test results.

Compound	Spiked mass level 1	Spiked mass level 2	Spiked mass level 3	Average R_an_	CV
Unit	μg	μg	μg	%	%
benzene	0.52	2.62	10.50	97.8	1.5
toluene	1.73	8.64	34.60	99.0	1.3
o-xylene	1.76	8.79	35.20	93.7	0.5
ethylbenzene	1.73	8.64	34.60	103.3	1.0
m- + p-xylene	3.44	17.20	68.90	99.6	0.7
isopropyl benzene	1.73	8.66	34.60	107.1	0.8
1,3,5-trimethybenzene	1.73	8.65	34.60	102.4	0.3
1,2,4-trimethybenzene	1.76	8.82	35.30	94.1	4.6
1,2,3-trimethybenzene	1.81	9.07	35.30	89.1	2.2
2-methylpentane	1.29	6.44	25.80	105.9	1.2
3-methylpentane	1.31	6.57	26.30	105.8	1.5
n-hexane	1.32	6.58	26.30	108.0	3.1
n-heptane	1.36	6.79	27.20	107.9	1.5
n-octane	1.41	7.05	28.20	104.6	6.2
n-nonane	1.43	7.17	26.70	109.6	0.9
n-decane	1.46	7.30	29.20	110.4	0.4
n-undecane	1.49	7.44	29.80	111.8	0.6
n-dodecane	1.51	7.54	30.20	116.6	2.4
cyclopentane	1.47	7.36	29.40	101.7	1.0
methylcyclopentane	1.61	8.05	32.20	105.5	1.1
cyclohexane	1.55	7.76	31.00	106.3	1.0
methylcyclohexane	1.54	7.68	30.70	107.4	1.2
tert-butyl methyl ether	1.49	7.46	29.80	101.7	3.2
tert-butyl ethyl ether	1.55	7.77	31.10	106.2	0.9

The concentration of the generated gas mixture delivered to the exposure chamber is verified by sampling onto activated charcoal tubes at 100 mL/min and subsequent determination of the benzene content of each tube by the MDHS96 method described in Health and Safety Executive (UK) (25).

Sampling was performed on six tubes during each 8-h experiment (three replicate tubes for the initial 4 h, three for the final 4 h). Sampling pumps used throughout this experimental study are Gilian LFS-113 low-flow air sampling pumps, manufactured by Sensidyne, Clearwater, Florida, United States, with operating flow range from 5 to 200 mL/min (constant flow mode). Charcoal tubes for all experiments were produced in-house by packing borosilicate glass tubes with two sections (front section, 350 mg; back, 150 mg) of activated charcoal for chromatography, particle size 0.3–0.5 mm (35–50 mesh), preconditioned by the manufacturer (the same used for RAD130 samplers). Analysis of activated charcoal tubes has been performed by transferring the charcoal substrate of each section to a glass tube, adding 2 mL of carbon disulfide and 100 μL of internal standard solution, vortexing for 30 s and leaving in contact for 30 min, then transferring the solution to autosampler vials (screw cap with PTFE lined septa, 2 mL), and submitting to HRGC-FID. The analytical instrumentation, chromatographic column, and operating parameters are the same as described before, regarding the analytical procedure of *radiello*® samplers. Results, expressed as average values over the six measurements, are discussed in the following section.

## Results

### Reference concentration, overall sampler uptake, leak test

The validation procedure for a diffusive sampler according to ISO 23320 standard is subject to some prerequisites concerning the reference concentration delivered to the exposure chamber. Target compound concentration has to be measured by an independent method to verify that the actual concentration (*C_meas_*) is within ±10% with respect to the expected one (*C_ref_*, which is calculated by the flow rate ratios in the dilution system, see ISO 23320 in paragraph 7.4.2.1 and 7.4.2.3). Benzene concentration values, measured by the independent method described before and compared to expected values, are displayed in Table 3, showing satisfactory agreement for all experiments performed at 20°C and relative humidity of 50%. When measuring at 20% relative humidity and low concentration (0.1 RV, 17.4 μg/m^3^), we observed a 21% difference between expected and measured concentration, and also when measuring at 80% relative humidity and high concentration (2 RV, 312 μg/m^3^), we observed a 12% difference between expected and measured concentration. Since no information is given, however, about method recovery at high or low humidity in the MDHS96 method described in Health and Safety Executive (UK) (25), or concerning measurement uncertainty component associated with relative humidity, we considered that good agreement between the target and measured concentration shown by experiments performed at 20°C and relative humidity of 50% demonstrates sufficiently accurate delivery of the target concentration to the exposure chamber in all cases. Reference concentration is therefore assumed to coincide with the expected value, derived from mass flow values controlled by the LNI Sonimix gas dilution apparatus, in all subsequent calculations.

**Table 3:** Benzene concentration in the exposure chamber (expected vs. measured by independent method).

Experiment	*T*	*RH*	*C_ref_*	*C_meas_*	*Δ*
Unit	°C	%	μg/m^3^	μg/m^3^	%
0.1 RV	20	50	18.5	17.8	−3.8
0.1 RV	20	50	18.9	18.1	−4.2
0.5 RV	20	50	65.7	59.4	−9.6
0.5 RV	20	50	66.1	62.3	−5.7
1 RV	20	50	145	130	−10.4
1 RV	20	50	144	132	−8.3
2 RV	20	50	281	264	−6.0
2 RV	20	50	284	272	−4.1
0.1RV	20	80	17.6	16.3	−7.4
0.1RV	20	20	17.4	13.8	−20.7
2 RV	20	80	312	275	−12.0
2 RV	20	20	311	296	−4.9
2 RV	40	50	282	290	2.9
2 RV	10	50	320	302	−5.4

The ISO 23320 standard requires also that the overall sampling uptake by all diffusive samplers, added to the sampling rate of the independent method (either by online measurement instrumentation or, as in the present case, by pumped sampling), shall not represent more than 75% of the overall gas flow delivered to the exposure chamber, in order to ensure concentration stability at the reference value and no “starvation” effect due to the presence of diffusive samplers. In the experimental set-up described here, the flow rate in the exposure chamber ranges from 15 to 26 L/min depending on the desired concentration. Assuming the lowest flow rate value (15 L/min) and the case of 24 RAD130 and three pumped samplers simultaneously present in an experiment, the combined uptake rate of diffusive samplers (for benzene 74.6 mL/min on RAD130) and sampling flow of pumped samplers (100 mL/min) sums to approximately 2.1 L/min. The combined flow rate of all samplers therefore is equal to 14% of the overall flow rate of the gaseous mixture delivered to the exposure chamber, so the prerequisite is fulfilled (14% < 75%).

Samplers used for workplace exposure monitoring, implying separate sampling and analytical steps (defined as “type A” samplers by the ISO 23320 standard), should demonstrate an analytical blank value, which is compatible with the monitoring purpose, i.e., less than the adsorbed mass of benzene at 0.1 RV for a sampling duration of 8 h, which corresponds to 573 ng (basing on experimentally measured uptake rate of the diffusive sampler, see the following paragraph). Actually, the blank value for the RAD130 sampler is less than the lower limit of quantitation (LLOQ) of the analytical procedure, which corresponds to 38 ng, so the requirement would be fulfilled even considering quite shorter exposure times, down to half an hour, or lower benzene exposure concentrations.

Packaged samplers should also retain the described blank value when transported to and from the monitoring site, or when temporarily stored in a potentially contaminated area, as can be the case during on-field sampling campaigns. For this purpose, the ISO 23320 standard requires that the sampler leak test is performed, by keeping six replicate unused samplers, still packaged, for 8 h in a test atmosphere at a concentration of approximately 2 RV (i.e., benzene at 320 μg/m^3^); six RAD130 samplers, packaged as they are usually shipped (with the ss cylinder packed with the activated charcoal substrate contained in the glass tube, firmly capped by the PE stopper, and the tube wrapped with a polyethylene thermo-welded foil) were exposed at benzene concentrations of 283 μg/m^3^, at 20.6°C average temperature and 49.7% average relative humidity for 473 min (which actually corresponds to twice the exposure time recommended by paragraph 8.2.3 of the ISO 23320 standard). Analysis of the six samplers showed no detectable amount of benzene, and as the LLOD for this substance corresponds to 13 ng, the requirement is fulfilled. The exposure chamber was actually fed with the gaseous mixture whose composition is shown in Table 1, properly diluted to obtain the described concentration of benzene and the corresponding concentrations of other VOCs (actual concentration values can be calculated by proportion to cylinder concentrations). No contamination by other VOCs than benzene is observed either.

### Nominal uptake rate

The nominal value of sampler uptake rate *U_d_* is obtained by exposure of six diffusive samplers for an exposure time *t_e_* of 4 h (251 min as tested) to a test atmosphere at concentration *ß_a_* corresponding to RV (actual value for benzene concentration *ß_a_* = 144.8 μg/m^3^), at the average temperature of 20°C and average relative humidity of 50% (actual values 21.4°C and 46.7%, respectively), as described in paragraph 8.2.1 of the ISO 23320 standard. After analytical determination of sampled mass *m_d_*, the nominal uptake rate is calculated for each replicate sampler according to Equation (1):


(1) $$U_{d}=\frac{m_{d}-m_{b}}{R_{\text{an}}\cdot {ß}_{a}\cdot t_{e}}$$

where *m_b_* is the blank value (usually negligible for RAD130) and *R_an_* represents the analytical recovery of benzene or each other compound for which the calculation is performed (this factor is already included if calibration is performed by the phase-equilibrium method). Results are shown in Table 4 (benzene sampled mass *m_d_*, reference exposure concentration *C_ref_*, exposure time *t_e_*, uptake rate *U_e_*, average uptake rate, and its coefficient of variation CV).

**Table 4:** Nominal uptake rate.

Sample	*m_d_*	*C_ref_*	*t_e_*	U_d_	U_d_	CV
Unit	μg	μg/m^3^	min	ml/min	ml/min	%
4 h @ 1 RV replicate 1	2.70	144.8	251	74.19	74.65	0.48
4 h @ 1 RV replicate 2	2.71	144.8	251	74.67		
4 h @ 1 RV replicate 3	2.72	144.8	251	74.72		
4 h @ 1 RV replicate 4	2.72	144.8	251	74.88		
4 h @ 1 RV replicate 5	2.73	144.8	251	75.14		
4 h @ 1 RV replicate 6	2.70	144.8	251	74.31		

### Storage after sampling and bias due to the selection of a non-ideal sorbent

The stability of samples upon storage has been studied as described in paragraph 8.3.1.3.1 of ISO 23320 standard, by exposing six sets of six replicate samples in the conditions shown in Table 5. As it is known that samples exposed to high humidity levels (both in ambient or workplace air) may show worse stability upon storage, the ISO 23320 standard requires to perform exposure at high humidity (80 ± 5%). Storage was performed in a refrigerator, free from solvents or other chemicals, at 4 ± 2°C. Two concentration levels were tested, corresponding to 0.1 RV and 2 RV, exposure time was 6 h. For each concentration level, 18 replicate RAD130 samplers were exposed, and then, 6 replicate samplers were analyzed within 24 h from exposure, 6 more replicate samplers were analyzed after 1 month, and the last 6 samples were analyzed after 2 months from exposure. The results are listed in Table 6 for the experiment at 0.1 OELV and at 2 RV (average benzene sampled mass *m_d_* recovered within each group of replicate samplers by analysis within 24 h, after 30 days and after 65 days, percentage difference upon storage). As stated in paragraph 6.3.1.4 of ISO 23320 standard, the mean value of the recovered mass after storage should not differ by more than 10% from the value before storage, this requisite is fulfilled for storage at 4 ± 2°C for up to 2 months (65 days).

**Table 5:** Storage test exposure conditions.

Experiment	*T*	*RH*	*C_ref_*	*t_e_*
Unit	°C	%	μg/m^3^	min
6 h @ 0.1 RV 80% RH	21.1	78.0	17.6	365
6 h @ 2 RV 80% RH	21.0	78.8	312.3	363

**Table 6:** Storage test, results upon exposure @ 0.1RV and 2 RV.

Experiment	Average *m_d_*	CV	Storage time	Δ
Unit	μg	%	Days	%
6 h @ 0.1 RV	0.477	0.70	≤ 1	
6 h @ 0.1 RV	0.436	3.0	30	−8.5
6 h @ 0.1 RV	0.440	1.0	65	−7.7
6 h @ 2 RV	8.137	0.45	≤ 1	
6 h @ 2 RV	7.629	4.3	30	−6.2
6 h @ 2 RV	7.936	0.90	65	−2.5

Diffusive samplers exposed to rapidly changing concentrations of the target chemicals can be affected by backdiffusion phenomena if a non-ideal sorbent is used. A test is therefore foreseen in the ISO 23320 standard in order to estimate the maximum bias that can be encountered in a real non-constant atmosphere, and the requisite (stated in paragraph 6.3.1.2 of the standard) is that bias measured in the experimental set-up described in the following should not be higher than 10%. Two sets of six replicate samplers were exposed for 45 min to a benzene concentration corresponding to 2 RV (actual value 290.5 μg/m^3^), then one set was removed from the atmosphere and the other was exposed for a further 430 min to pure air. During the whole experiment, average temperature and relative humidity were 20.7°C and 77.1%, respectively. The two sample sets were then analyzed, and the results are shown in Table 7 (sampled mass for each replicate sampler, average sampled mass for the two groups, percentage difference). The difference between the means is 2.1%, and the requirement is then fulfilled. Moreover, Student’s *t*-test has been applied to the two populations (calculations not shown). The null hypothesis is zero difference between the two means, the significance value is 0.05, and the chosen *t*-test is the two-tail case with unequal variance within the two populations. The result is as follows: *t*-stat is equal to −2.1885 and comprised between – t critical and + t critical (−2.3060 < −2.1885 < 2.3060); therefore, the null hypothesis cannot be rejected on the basis of the experimental data. We can therefore conclude that the average benzene adsorbed mass is not significantly different between the two groups of samplers (exposed, or not, to zero air).

**Table 7:** Backdiffusion test results.

Sample	*m_d_*	Average *m_d_*	Sample	*m_d_*	Average *m_d_*	Δ
Unit	μg	μg	Unit	μg	μg	%
45′ @ 2 RV replicate 1	0.942	0.936	45′ @ 2 RV + 7.5 h @ zero air replicate 1	0.960	0.956	2.1
45′ @ 2 RV replicate 2	0.915		45′ @ 2 RV + 7.5 h @ zero air replicate 2	0.968		
45′ @ 2 RV replicate 3	0.963		45′ @ 2 RV + 7.5 h @ zero air replicate 3	0.938		
45′ @ 2 RV replicate 4	0.952		45′ @ 2 RV + 7.5 h @ zero air replicate 4	0.951		
45′ @ 2 RV replicate 5	0.929		45′ @ 2 RV + 7.5 h @ zero air replicate 5	0.965		
45′ @ 2 RV replicate 6	0.916		45′ @ 2 RV + 7.5 h @ zero air replicate 6	0.953		

Exposure at 2 RV for 45 min yields an average benzene sampled mass of 0.936 μg, corresponding to a measured concentration of 278.6 μg/m^3^ (by calculations based upon the nominal uptake rate value of 74.65 mL/min). The ratio between measured and reference concentration (290.5 μg/m^3^) yields a method recovery of 0.959, and a method bias of 2.2% (see following paragraphs for a discussion of these parameters). Comparing method recovery and method bias calculated for this experiment with the overall validation data shown in the following, we observe that the proposed sampling and analytical method is fit for the purpose of short time duration measurements.

### Method recovery and method precision

Once the nominal uptake rate of the sampling system for a target compound has been experimentally measured, several tests are envisaged by the ISO 23320 standard to study method recovery, method precision, and their variability upon the effect of sampling parameters, such as exposure time, exposure concentration, temperature, and relative humidity of the test atmosphere. Within each experiment, a minimum of six replicate samplers are exposed to the target substance (or, as in the present case, to benzene and the other VOCs listed in Table 1) in a definite set of experimental conditions (exposure time *t_e_*, reference concentration *C_ref_*, temperature *T*, and relative humidity *RH*). The samplers are analyzed to measure sampled mass *m_d_*, and then, exposure concentration *ß_a_* is calculated by using the nominal uptake rate according to Equation (2):


(2) $${ß}_{a}=\frac{m_{d}-m_{b}}{R_{\text{an}}\cdot U_{d}\cdot t_{e}}$$

where *t_e_* is exposure time, *m_b_* is the blank value (usually negligible for RAD130), and *R_an_* represents the analytical recovery of benzene or each other compound for which the calculation is performed (this factor is already included if calibration is performed by the phase-equilibrium method). For each exposure combination of sampling parameters, the measured concentration is calculated for each replicate diffusive sampler. Each measured concentration value is then divided by the reference concentration of the test atmosphere to obtain method recovery MR: ideally, the ratio should be close to 1 in all cases, if the uptake rate is not influenced by sampling parameters. For each group of replicate samplers, the mean method recovery and its coefficient of variation are calculated, and these data will constitute the basis for the estimation of method uncertainty, as will be described in the following paragraph.

### Effect of exposure time

Three groups of six replicate diffusive samplers RAD130 were exposed in the experimental conditions displayed in Table 8, test # 1, 2, and 3: benzene concentration corresponding to 1 RV (i.e., close to 160 μg/m^3^), temperature 20°C and 50% relative humidity, and exposure time of 1 h, 4 h, and 8 h. The samples were analyzed to determine benzene content; for each sample, exposure concentration was calculated and compared with reference concentration to obtain method recovery. For each exposure duration, the average method recovery and the corresponding CV were calculated, and the results are shown in Table 9, tests # 1, 2, and 3. Then, the overall method recovery for exposure durations from 1 to 8 h was obtained, and it is equal to 1.015 (CV = 1.3%).

**Table 8:** Effect of exposure time, exposure concentration, relative humidity, and temperature–test conditions.

Test #	Conditions	*T*	*RH*	*C_ref_*	*t_e_*
Unit		°C	%	μg/m^3^	min
1	1 h @ 1 RV	21.1	49.6	144.3	60
2	4 h @ 1 RV	21.4	46.7	144.8	251
3	8 h @ 1 RV	21.3	48.4	144.6	462
4	4 h @ 0.1 RV	21.4	49.2	18.5	238
5	4 h @ 0.5 RV	21.2	51.5	65.7	243
6	4 h @ 1 RV	21.4	46.7	144.8	251
7	4 h @ 2 RV	20.4	48.4	281.4	248
8	6 h @ 0.1 RV and 80% RH	21.1	78.0	17.6	365
9	6 h @ 0.1 RV and 20% RH	20.2	17.8	17.4	362
10	6 h @ 2 RV and 80% RH	21.0	78.8	312.3	363
11	6 h @ 2 RV and 20% RH	20.3	21.9	310.9	370
12	6 h @ 2 RV 38°C	38.0	47.4	282.1	363
13	6 h @ 2 RV 11°C	11.4	48.6	319.7	364

**Table 9:** Effect of exposure time, exposure concentration, relative humidity, and temperature–test results.

Test #	Conditions	Average *m_d_*	CV	*t_e_*	*ß_a_*	*C_ref_*	MR	Average MR
Unit		μg	%	min	μg/m^3^	μg/m^3^		
1	1 h @ 1 RV	0.662	0.42	60	147.8	144.3	1.024	1.015
2	4 h @ 1 RV	2.714	0.48	251	144.8	144.8	1.000	
3	8 h @ 1 RV	5.086	0.71	462	147.5	144.6	1.020	
4	4 h @ 0.1 RV	0.341	1.7	238	19.2	18.5	1.036	1.015
5	4 h @ 0.5 RV	1.195	1.4	243	65.9	65.7	1.002	
6	4 h @ 1 RV	2.714	0.48	251	144.8	144.8	1.000	
7	4 h @ 2 RV	5.311	0.94	248	286.9	281.4	1.020	
8	6 h @ 0.1 RV and 80% RH	0.477	0.70	365	17.5	17.6	0.994	1.010
9	6 h @ 0.1 RV and 20% RH	0.483	2.1	362	17.9	17.4	1.028	
10	6 h @ 2 RV and 80% RH	8.137	0.45	363	300.3	312.3	0.961	
11	6 h @ 2 RV and 20% RH	9.077	0.96	370	328.6	310.9	1.057	
12	6 h @ 2 RV 38°C	8.909	1.2	363	300.6	282.1	1.066	1.044
13	6 h @ 2 RV 11°C	8.883	1.7	364	326.9	319.7	1.023	

### Effect of exposure concentration

Four groups of six replicate diffusive samplers RAD130 were exposed in the experimental conditions displayed in Table 8, test # 4, 5, 6, and 7: exposure time of 4 h, temperature 20°C and 50% relative humidity, and benzene concentration corresponding to 0.1 RV–0.5 RV–1 RV–2 RV (i.e., close to 16, 80, 160, and 320 μg/m^3^). The samples were analyzed to determine benzene content; for each sample, benzene exposure concentration was calculated and compared with reference concentration to obtain method recovery. For each exposure concentration level, the average method recovery and the corresponding CV were calculated; the results are shown in Table 9, tests # 4, 5, 6, and 7. Then, overall method recovery for exposure concentrations from 0.1 to 2 RV (i.e., from 16 to 320 μg/m^3^) was obtained, which is also equal to 1.015 (but with CV = 1.7%).

### Effect of relative humidity of the test atmosphere

Four groups of six replicate diffusive samplers RAD130 were exposed in the experimental conditions displayed in Table 8, tests # 8, 9, 10, and 11: exposure time of 6 h, temperature 20°C, benzene concentration corresponding to 0.1 RV and 2 RV (i.e., close to 16 and 320 μg/m^3^), and relative humidity close to 20 and 80% (four experimental set-up combinations overall). The samples were analyzed to determine benzene content; for each sample, benzene exposure concentration was calculated and compared with reference concentration to obtain method recovery. For each exposure combination of concentration and relative humidity conditions, average method recovery and the corresponding CV were calculated, and results are shown in Table 9, tests # 8, 9, 10, and 11. Then, the difference was calculated between the mean average recovery values at 80 and 20% relative humidity for each concentration level, to estimate the effect of relative humidity on method recovery according to paragraph 8.3.3.4 of ISO 23320 standard. At 0.1 RV (benzene concentration of approximately 17 μg/m^3^) average method recovery was 99.4% at 80% relative humidity, and 103% at 20% relative humidity; the difference is equal to 3.4%. At 2 RV (benzene concentration of approximately 320 μg/m^3^) average method recovery was 96.1% at 80% relative humidity, and 106% at 20% relative humidity; the difference is equal to 9.0%. The ISO 23320 standard requires to consider the maximum observed difference (in the present case, measured at 2 RV) in the estimation of measurement uncertainty contribution due to the effect of humidity, as will be described in the following paragraph.

### Effect of temperature of the test atmosphere

Two groups of six replicate diffusive samplers RAD130 were exposed in the experimental conditions displayed in Table 8, tests # 12 and 13: exposure time of 6 h, benzene concentration corresponding to 2 RV (i.e., close to 320 μg/m^3^), and relative humidity of 50%, temperature of approximately 40°C and 10°C (two experimental set-up combinations overall). The samples were analyzed to determine benzene content, and for each exposure combination, average method recovery and the corresponding CV were calculated; then, exposure concentration was calculated and compared with reference concentration to obtain method recovery; results are shown in Table 9, tests # 12 and 13. Then, the difference was calculated between the mean average recovery values at 40°C and 10°C, to estimate the effect of temperature on method recovery according to paragraph 8.3.3.5 of ISO 23320 standard. The average method recovery was 106.6% at 38°C and 102.3% at 11°C; the difference corresponds to 4.1% with respect to overall method recovery.

### Uncertainty of measurement

All random and non-random uncertainty components of the measuring procedure have been identified by constructing a cause-and-effect diagram, as described in the Eurachem/CITAC Guide “Quantifying Uncertainty in Analytical Measurement” (26); then, following the approach suggested in Annex B of the ISO 23320 standard, the estimation of uncertainty of measurement was accomplished.

The present method, for the measurement of benzene (and other VOCs) concentration in workplace atmospheres, involves two major steps: sampling and analysis. The combined standard uncertainty u_c_, expressed as a percentage, is calculated according to Equations (3–5):


(3) $$u_{s}=\sqrt{u_{s_{r}}^{2}+u_{s_{nr}}^{2}}$$

(4) $$u_{a}=\sqrt{u_{a_{r}}^{2}+u_{a_{nr}}^{2}}$$

(5) $$u_{c}=\sqrt{u_{s}^{2}+u_{a}^{2}}$$

where

u_s_ = sampling uncertainty

u_sr_ = random sampling uncertainty

u_snr_ = non-random sampling uncertainty

u_a_ = analytical uncertainty

u_ar_ = random analytical uncertainty

u_anr_ = non-random analytical uncertainty

The expanded uncertainty of the measuring procedure *U*, expressed as a percentage, is calculated using a coverage factor *k* = 2 (see ISO 23320, paragraphs 8.4.2.6 and 8.4.3) according to Equation (6):


(6) $$U=2\cdot u_{c}$$

The following contributions due to sampling and analytical steps have been considered for uncertainty estimation: concerning the sampling, uncertainty associated with mass uptake, sampling efficiency, and sample storage; concerning analysis, uncertainty associated with method recovery, method variability, calibration, and response drift.

### Uncertainty associated with mass uptake (uptake rate and sampling time)

For diffusive sampling, mass uptake has the following sources of uncertainty: uptake rate and sampling time.

The random and non-random uncertainty components associated with the uptake rate have been estimated from the results of the replicate samples collected from the test atmosphere experiment carried out to determine the nominal uptake rate (data displayed in Table 4).

The random and non-random uncertainty components associated with the uptake rate are given by Equation B1 (for the definition of each term, see Annex B of ISO 23320 standard, paragraph B.2.2):


(B1) $$u_{ur}=\sqrt{\frac{{\left(K_{v,r}\right)}^{2}}{n}+{\left(u_{rc}\right)}^{2}}$$

In our experiment, the number of replicate samples *n* is 6 and the coefficient of variation *K_v,r_* is 0.48%. The uncertainty associated with the reference concentration of the test atmosphere *u_rc_* has been calculated by considering the uncertainty associated with benzene concentration in the cylinder gas mixture and the uncertainty associated with mass flow measurements within the dilution system used to feed the exposure chamber, the result is *u_rc_* = 3.6% from 0.1 to 2 RV (i.e., from 16 to 320 μg/m^3^). Applying Equation (B1), we obtain that uncertainty associated with the uptake rate *u_ur_* is equal to 3.6%.

Sampling time is recorded to the nearest minute, so the maximum bias is 0.5 min at both the start and end of the experiment. The shortest experiments we performed imply a sampling duration of 60 min; by summing the maximum biases and dividing by 60 min and by √6 (assuming a triangular probability distribution), we obtain that uncertainty associated with sampling time is 0.7%.

### Uncertainty associated with sampling efficiency (back diffusion and exposure time)

Following the approach outlined in paragraph B.3.1 in Annex B of the ISO 23320 standard, and assuming a rectangular probability distribution, the uncertainty associated with back diffusion *u_bd_* is given by *Δ_bd_* /√3 where *Δ_bd_* is the difference, in percent, between the mean results of replicate samples listed in Table 7, equal to 2.1%. The uncertainty associated with back diffusion is therefore equal to 1.2%.

The non-random uncertainty component associated with exposure time has been estimated, as described in paragraph B.3.2 in Annex B of the ISO 23320 standard, by the analysis of replicate samples listed in Table 9, tests # 1, 2, and 3 (exposure time 1, 4, and 8 h; atmosphere concentration 1 RV). Assuming a rectangular probability distribution, the uncertainty associated with exposure time *u*_te_ is given by *u*_te_ = *Δ*_te_/√3 where *Δ*_te_ is the highest difference between the mean results of replicate samples collected from test atmospheres at different exposure times, in percent. In the present case, the highest difference has been observed between the mean concentrations measured at 1 RV upon sampling for 4 h and 1 h, equal to 2.4%. The non-random uncertainty component associated with exposure duration is therefore equal to 1.4%.

### Uncertainty associated with sample storage

The non-random uncertainty component associated with sample storage has been estimated, as described in paragraph B.4 in Annex B of the ISO 23320 standard, by the analysis of replicate samples listed in Table 6 (exposure time 6 h; atmosphere concentration 0.1 RV and 2 RV; relative humidity 80%; analysis within 24 h, after 30 days and after 65 days). Assuming a rectangular probability distribution, the uncertainty associated with sample storage *u*_st_ is given by equation *u*_st_ = *Δ*_st_/√3 where *Δ*_st_ is the difference between the mean results of replicate samples analyzed immediately after sampling and replicate samples analyzed after the maximum storage time, in percent. In the present case, the difference (for storage of 65 days at a temperature of 4°C, in a clean refrigerator, free from solvents or other volatile organic compounds) is equal to 7.7% at 0.1 RV and 2.5% at 2 RV. The worst-case non-random uncertainty component associated with sample storage, corresponding to the experiment at 0.1 RV, is therefore equal to (7.7/√3) %, which is 4.4%.

### Uncertainty associated with method recovery

The experimental data collected when carrying out the tests whose results are displayed in Table 9, tests # from 1 to 13, give representative information about the factors causing variation and bias (relative to a reference concentration value) that occur in routine applications of the specified method of measurement, such as exposure time, concentration, relative humidity, and temperature. The uncertainty associated with exposure time has been already estimated; in the present section, we will show how uncertainty contributions due to concentration (method bias), relative humidity, and temperature are estimated.

Method bias is calculated from the results of the replicate samplers collected upon exposure for 4 h at 50% relative humidity, 20°C temperature, and benzene concentration of 0.1 RV, 0.5 RV, and 2 RV (see Table 8, tests # 4, 5, and 7).

For each concentration value, the non-random uncertainty associated with the method bias *u_mb_* is calculated according to Equation B2 (for the definition of each term see the Annex B of ISO 23320 standard, paragraph B.5.3):


(B2) $$u_{mb}=\sqrt{{\left(\frac{B_{m}}{k}\right)}^{2}+\frac{{\left(K_{v,rm}\right)}^{2}}{n}+{\left(u_{rc}\right)}^{2}}$$

In our experiment, the number of replicate samples n is 6 and the coverage factor k used in the calculation of the expanded uncertainty is 2. Method bias *B_m_* and coefficient of variation *K_v,rm_* are calculated at each level from the experimental data shown in Table 9, tests # 4, 5, and 7. The uncertainty associated with the reference concentration of the test atmosphere *u_rc_* has already been considered within uncertainty associated with uptake rate (see Equation B1), method bias at each concentration level is therefore calculated according to Equation B3 and displayed in Table 10.


(B3) $$u_{mb}=\sqrt{{\left(\frac{B_{m}}{2}\right)}^{2}+\frac{{\left(K_{v,rm}\right)}^{2}}{n}}$$

In order to calculate the expanded uncertainty of the measurement procedure, we consider the worst-case estimate, corresponding to 1.9% at 0.1 RV, to be valid in the entire applicability range.

**Table 10:** Estimation of method bias.

Test #	Conditions	*C_ref_*	*B_m_*	*K_v,rm_*	*u_mb_*
Unit		μg/m^3^	%	%	%
4	4 h @ 0.1 RV	18.5	3.6	1.7	1.9
5	4 h @ 0.5 RV	65.7	0.22	1.4	0.57
7	4 h @ 2 RV	281.4	2.0	0.94	1.1

The non-random uncertainty component associated with the effect of humidity has been estimated from the difference between the mean results of replicate samples collected from the test atmospheres at 80 and 20% relative humidity, and at 0.1 RV and 2 RV (data displayed in Table 9, tests # 8, 9, 10, and 11). At 0.1 RV, the difference between the means at 20 and 80% relative humidity is 3.4%; at 2 RV, the difference between the means at 20 and 80% relative humidity is 9.0%. Assuming a rectangular probability distribution, the uncertainty associated with the effect of humidity *u_h_* is given by *Δ_h_*/√3 where *Δ_h_* is the higher of the previously defined differences, that is 9.0%. The worst-case non-random uncertainty component associated with humidity is therefore equal to 5.2%.

The non-random uncertainty component associated with the effect of temperature has been estimated from the difference between the mean results of replicate samples collected from the test atmospheres at temperatures of 10°C and 40°C (actually, at 11.4 and 38.0°C), and at concentrations of 2 RV (data displayed in Table 9, tests # 12 and 13). Assuming a rectangular probability distribution, the uncertainty associated with the effect of temperature *u_T_* is given by *Δ_T_*/√3 where *Δ_T_* is the difference between the mean results of replicate samples collected from the described test atmospheres, equal to 4.1%. The uncertainty component associated with the effect of temperature is equal to 2.4%.

### Uncertainty associated with method variability

Method precision is calculated from the results of the replicate samples collected upon exposure for 4 h in test atmospheres at 0.1 RV, 0.5 RV, 1 RV, and 2 RV, with relative humidity of 50% and temperature of 20°C (data displayed in Table 9, test # 4, 5, 6 and 7). The random uncertainty component *u_mp_* is calculated according to Equation B4, for the definitions of involved terms and calculation of *K_vp,r_* see paragraph B.6.2 of the ISO 23320 standard.


(B4) $$u_{mp}=\sqrt{{\left(K_{v,m}\right)}^{2}+\left(1-\frac{1}{n}\right)\cdot {\left(K_{vp,r}\right)}^{2}}$$

Since for each experiment, a set of six samplers has been exposed, *n* = 6, and the term Kvp,r is calculated according to Equation B5:


(B5) $$K_{vp,r}=\sqrt{\frac{\left[{\left(K_{v,1}\right)}^{2}\right]+\left[{\left(K_{v,2}\right)}^{2}\right]+\left[{\left(K_{v,3}\right)}^{2}\right]+\left[{\left(K_{v,4}\right)}^{2}\right]\;}{4}}$$

where *K_v,1_*, *K_v,2_, K_v,3_*, and *K_v,4_* are the coefficients of variation at the four tested concentrations, in percent, and *K_v,m_* is the coefficient of variation of the means, in percent. The results are shown in Table 11, and *u_mp_* is equal to 2.01% over the measurement range from 0.1 to 2 RV (i.e., from 16 to 320 μg/m^3^).

**Table 11:** Estimation of method precision.

Test #	Conditions	*C_ref_*	*K_v_*	MR	Average MR	*K_v,m_*	*K_v,pr_*	*u_mp_*
Unit		μg/m^3^	%	%	%	%	%	%
4	4 h @ 0.1 RV	18.5	1.7	1.04	1.015	1.67	1.22	2.01
5	4 h @ 0.5 RV	65.7	1.4	1.00				
6	4 h @ 1 RV	144.8	0.48	1.00				
7	4 h @ 2 RV	281.4	0.94	1.02				

### Uncertainty associated with the analytical procedure

Uncertainty contribution associated with calibration solutions has been calculated within the validation procedure of the analytical method by applying a metrological approach to the procedure for preparing the calibration solutions (by weighing and dilution, using a calibrated balance with extended uncertainty of 0.14 mg in the measurement range around 20 g, and calibrated glassware with volume tolerances of ±0.4% for flasks and ± 0.8% for pipets). We obtain that the uncertainty contribution associated with the preparation of calibration solutions is equal to 1.7%.

The random uncertainty component associated with the calibration function is calculated from parameters obtained by applying the least-squares linear (or second-order polynomial) regression, as outlined in Ellison and Williams (26). It varies as a function of the sampled mass value; therefore, combined analytical uncertainty also varies with sampled mass value in the applicability range; data are shown in Table 12 (including the uncertainty component due to instrumental response drift, described hereby in the following).

**Table 12:** Combined analytical uncertainty.

Benzene mass	μg	0.10	0.20	0.30	0.40	0.50	1.00	1.31	5.24	10.48
*u_c_*	%	12.4	6.8	5.1	4.3	3.9	3.3	3.2	3.1	3.1

Instrument response drift is considered by introducing a 5% maximum instrument response drift allowance (*d_max_*) before recalibration. Quality control standards are injected every 20 samples (after the complete calibration curve, injected once a month if QC requisites are met). The non-random uncertainty component due to instrumental drift *u_dr_*, therefore, is estimated by assuming a rectangular probability distribution and corresponds to *u_dr_* = *d_max_* /√3, which is equal to 2.9%.

As benzene sampled mass upon exposure of RAD130 sampler at 0.1 RV (i.e., 16 μg/m^3^) for 8 h corresponds to 0.57 μg, and obviously exposure for 8 h at higher concentrations will increase sampled mass, we consider that maximum analytical uncertainty to be taken into account for 8-h measurements in the studied range between 0.1 and 2 RV is 3.9%, as can be seen in Table 12. This value will be introduced in the following calculations.

Analytical precision can be estimated using repeatability data, obtained by injecting replicate samples at different concentration levels within the method applicability range. However, as method variability is estimated according to equation (B4), the contribution of the analytical precision is already included as explained in paragraph B.6.7.1 of the ISO 23320 standard.

### Calculation of combined standard uncertainty and expanded uncertainty

To calculate the random and non-random components of sampling and analytical uncertainty, the relevant individual uncertainty components are combined according to Equations (3–5), and then, the expanded uncertainty of the measuring procedure is calculated by using a coverage factor of 2 as outlined in Equation (6). The components to be included in the calculations have been previously described and are summarized in Table 13. We can therefore calculate random and non-random contributions to sampling uncertainty, and by combining random and non-random contributions, we obtain that uncertainty associated with sampling is as follows:


$$u_{\text{sr}}=\sqrt{3.6^{2}+0.7^{2}+2.0^{2}}=4.2\%$$

$$u_{snr}=\sqrt{3.6^{2}+1.2^{2}+1.4^{2}+4.4^{2}+1.9^{2}+5.2^{2}+2.4^{2}}=8.5\%$$

$$u_{s}=\sqrt{4.2^{2}+8.5^{2}}=9.5\%$$

By combining sampling and analytical uncertainty contributions, we obtain


$$u_{c}=\sqrt{9.5^{2}+3.9^{2}}=10.3\%$$

By applying a coverage factor of 2, we obtain expanded uncertainty:


$$U_{e}=20.6\%$$

**Table 13:** Uncertainty components.

Description	Random	Non-random
Unit	%	%
Sampled air volume		
Uptake rate	3.6	
Reference concentration		3.6
Sampling time	0.7	
Sampling efficiency		
Back diffusion		1.2
Effect of exposure time		1.4
Storage		4.4
Method recovery		
Method bias		1.9
Effect of relative humidity		5.2
Effect of temperature		2.4
Method precision	2.0	

### Other gasoline VOC components: determination of nominal uptake rate, study of method performances, uncertainty calculations

The above-described method can be applied to determine the air concentration of several VOC gasoline components. The same experimental approach was applied as described for benzene, which allowed the determination of uptake rate values and method performances depending on sampling parameters (exposure time, exposure concentration, temperature and relative humidity of the atmosphere, sample storage, and back diffusion). Method uncertainty has been calculated by following the same procedures described above for benzene regarding other gasoline VOC components usually found in the same workplace environments, the results (uptake rate and its coefficient of variation, expanded uncertainty to be associated with the sampling and analytical method, and studied concentration range) are summarized in Table 14. The listed compounds have shown very good recovery in the back diffusion test (difference between sampled mass upon exposure for 30 min at high concentration only or for 30′ at high concentration and for 7.5 h to clean air were well within 10%, as required by the ISO 23320 standard, and in most cases lower than 5%) and in the storage test for 2 months (difference between sampled mass quantified upon analysis within 24 h from exposure and after 2 months from exposure lower than 10%, as required by the ISO 23320 standard, and in most cases well below 5%). The concentration range is generally similar to the one studied for benzene, with the exception of less volatile compounds (isopropyl benzene and the isomers of trimethylbenzene), which are present in the gaseous mixture used for feeding the exposure chamber at lower concentrations in order to avoid condensation within the gas cylinder. In all cases, the concentration range is quite low with respect to the present OELVs (where defined) but comparable to the studied benzene concentration range (from 16 to 320 μg/m^3^).

**Table 14:** Method performance for other gasoline components.

Compound	Nominal uptake rate	CV	*U_e_*	Studied concentration range
Unit	ml/min	%	%	μg/m^3^
methyl tertiary butyl ether	67.0	1.8	27.5	17–270
cyclopentane	72.4	0.95	55.3	14–215
2-methylpentane	65.0	1.5	30.1	17–269
3-methylpentane	65.5	0.97	21.9	17–266
n-hexane	64.7	1.1	18.1	17–260
methylcyclopentane	67.0	0.90	19.0	27–418
cyclohexane	66.5	0.80	19.5	18–281
n-heptane	59.2	0.92	18.5	20–302
methylcyclohexane	62.2	0.80	18.8	21–325
toluene	70.3	1.4	25.3	18–282
n-octane	54.1	1.4	21.0	22–341
ethylbenzene	65.6	1.5	28.1	20–310
m- + p-xylene	64.7	1.4	32.6	41–627
o-xylene	62.6	1.5	34.7	21–320
n-nonane	46.0	2.0	37.6	24–372
isopropyl benzene	60.2	2.0	36.0	4.0–62
1,2,3-trimethylbenzene	56.4	1.8	153	4.2–65
1,2,4-trimethylbenzene	54.5	1.8	125	4.8–74
1,3,5-trimethylbenzene	56.4	1.7	75.2	4.5–69

## Conclusion

A sampling and analytical method has been validated that allows workplace monitoring of benzene covering the low concentration range (from 0.005 to 0.1 ppm, 16 to 320 μg/m^3^) over an 8-h exposure duration, tested according to the proper and most recent European standard, EN ISO 23320:2022–“*Workplace air–Gasses and vapors–Requirements for evaluation of measuring procedures using diffusive samplers.”* The proposed method is fit for the generation of high-quality data concerning personal monitoring in a low-dose occupational exposure range in the chemical, petrochemical, foundry, and pharmaceutical industries, whereas previous studies were based on extrapolated data.

The relative extended uncertainty of the sampling and analytical method is equal to 20.6% over the entire studied range; therefore, the method is fit for benzene exposure measurements in workplaces where its concentration is expected to be in the low ppb range, according to the performance criteria established by the EN 482:2021–“*Workplace exposure–General requirements for the performance of procedures for the measurement of chemical agents”* standard.

The method relies upon a diffusive sampling technique, which ensures simplicity of use for industrial hygiene and HSE operators and intrinsic safety in potentially explosive atmospheres. The samplers are very lightweight and negligibly encumber the operators even when busy with demanding tasks in the production plants. Several monitoring tasks can be accomplished at a time, both by personal or fixed area sampling, and they can be performed during the usual 8-h shifts or for longer time durations, for example, for 12-h working shifts, with no limitations deriving from power supply availability, even in remote locations. The method is fit for purpose also for exposure monitoring over short time durations, aimed at evaluating personal exposure associated with specific tasks, or at investigating dose-rate responsiveness in order to understand which dose metrics (cumulative or peak exposures) are relevant in hazard assessments.

The collected samples have been demonstrated to be stable upon storage for up to 65 days, this result is particularly important if we consider that an industrial hygiene survey in a production plant may require several weeks (1 to 3, reasonably) to cover all shifts and all similar exposure groups (SEGs). All samples can be stored together until the survey is complete, and then shipped to the laboratory and analyzed within 2 months from the beginning of the monitoring campaign.

The methodology validated in this study will be integral to the monitoring of benzene in industrial and professional gasoline exposure environments to assure compliance with current and future acceptably safe levels. This validated technique can also be used to measure other VOC gasoline components, as regulatory science continues to advance safe exposure considerations to some of these, including isopropyl benzene (also known as 2-phenylpropane or cumene) (27, 28) and n-hexane (29), measured in this study, and potentially similar other chemicals including 2-phenylpropene (28) (also known as alpha-methyl styrene, CAS number 98–83-9), not measured within this study but eligible for sampling and analysis by the same technique.

## Data availability statement

The original contributions presented in the study are included in the article/supplementary material, further inquiries can be directed to the corresponding author.

## Author contributions

LZ: Conceptualization, Data curation, Formal analysis, Investigation, Project administration, Resources, Supervision, Validation, Visualization, Writing – original draft. CB: Investigation, Resources, Writing – review & editing. RS: Formal analysis, Investigation, Writing – review & editing. GH: Visualization, Writing – review & editing. EG: Writing – review & editing. DC: Writing – review & editing.

## Footnotes

^1^Additional exposure experiments at benzene concentration up to 1 mg/m^3^ have been carried out, which will be described elsewhere, demonstrating fit-for-purpose method recovery (by comparison with pumped sampling) and reproducibility.
